# Auto-acetylation on K289 is not essential for HopZ1a-mediated plant defense suppression

**DOI:** 10.3389/fmicb.2015.00684

**Published:** 2015-07-08

**Authors:** José S. Rufián, Ainhoa Lucía, Alberto P. Macho, Begoña Orozco-Navarrete, Manuel Arroyo-Mateos, Eduardo R. Bejarano, Carmen R. Beuzón, Javier Ruiz-Albert

**Affiliations:** Departamento Biología Celular, Genética y Fisiología, Instituto de Hortofruticultura Subtropical y Mediterránea “La Mayora" – Universidad de Málaga – Consejo Superior de Investigaciones CientíficasMálaga, Spain

**Keywords:** type III secretion system, effector, ETI, suppression, plant defense, SAR, acetylation, *Pseudomonas syringae*

## Abstract

The *Pseudomonas syringae* type III-secreted effector HopZ1a is a member of the HopZ/YopJ superfamily of effectors that triggers immunity in *Arabidopsis*. We have previously shown that HopZ1a suppresses both local [effector-triggered immunity (ETI)] and systemic immunity [systemic acquired resistance (SAR)] triggered by the heterologous effector AvrRpt2. HopZ1a has been shown to possess acetyltransferase activity, and this activity is essential to trigger immunity in *Arabidopsis*. HopZ1a acetyltransferase activity has been reported to require the auto-acetylation of the effector on a specific lysine (K289) residue. In this paper we analyze the relevance of autoacetylation of lysine residue 289 in HopZ1a ability to suppress plant defenses, and on the light of the results obtained, we also revise its relevance for HopZ1a avirulence activity. Our results indicate that, while the HopZ1a^K289R^ mutant is impaired to some degree in its virulence and avirulence activities, is by no means phenotypically equivalent to the catalytically inactive HopZ1a^C216A^, since it is still able to trigger a defense response that induces detectable macroscopic HR and effectively protects *Arabidopsis* from infection, reducing growth of *P. syringae* within the plant. We also present evidence that the HopZ1a^K289R^ mutant still displays virulence activities, partially suppressing both ETI and SAR.

## Introduction

Many gram-negative pathogenic bacteria use a type III secretion system (T3SS) to secrete proteins, known as effectors, directly inside the host cell cytosol. Type III effectors (T3Es) modulate diverse processes inside the host, suppressing plant defense responses triggered upon recognition of the pathogen (Gohre and Robatzek, 2008). One such defense is triggered upon recognition of conserved pathogen-associated molecular patterns (PAMPs) and is known as PTI (Boller and Felix, 2009). T3Es can be directly or indirectly detected by the plant resistance proteins, triggering a second line of defense, a strong response known as ETI that is typically accompanied by a type of programmed cell death referred to as the hypersensitive response (HR). The ETI response determines a severe restriction in pathogen growth (Chisholm et al., 2006). Effectors triggering strong immunity were originally named avirulence factors, as their expression by a pathogen determines resistance against the disease (Mansfield, 2009).

Effectors can also suppress ETI, cell death and other HR-associated phenomena, thus promoting pathogen growth and the development of disease (Jones and Dangl, 2006). We have shown that HopZ1a from *Pseudomonas syringae* pv. *syringae* is one such effector (Macho et al., 2010). Heterologous expression of HopZ1a from *P. syringae* pv. *tomato* DC3000 (hereafter DC3000), suppresses RNA and protein accumulation of *PR1*, triggered in *Arabidopsis* by this pathogen (Macho et al., 2010), and partially suppresses the ETI triggered by the expression of the heterologous effectors AvrRpt2, AvrRps4, and AvrRpm1 (Macho et al., 2010). These defense suppression activities of HopZ1a are similar to those described for the related *Xanthomonas* effector AvrBsT (Kim et al., 2010, 2013; Szczesny et al., 2010). We have also demonstrated that HopZ1a is capable of suppressing systemic acquired resistance (SAR) triggered by either virulent or avirulent bacteria (Macho et al., 2010). All these virulence activities are fully dependent on HopZ1a C216 catalytic residue. In turn, HopZ1a triggers SA and EDS1-independent immunity in *Arabidopsis* (Lewis et al., 2010; Macho et al., 2010) upon recognition by the *ZAR-1* resistance gene (Lewis et al., 2010).

HopZ1a is a member of the YopJ/HopZ effector superfamily, whose members share a conserved catalytic triad (C/H/D) and have been shown to perform numerous biochemical activities, mainly as proteases and/or acetyltransferases, with some effectors such as YopJ displaying up to three different biochemical functions concurrently (Orth et al., 2000; Mukherjee et al., 2006; Sweet et al., 2007). To explain such multiplicity of activities, it has been suggested that acetyltransferases and proteases might use the same catalytic mechanism on different substrates (Mukherjee et al., 2007). HopZ1a has been described to display cysteine protease activity (Ma et al., 2006), but also acetyltransferase activity on a number of plant target proteins, with the latter activity requiring the plant cofactor phytic acid (Lee et al., 2012; Jiang et al., 2013; Lewis et al., 2013). HopZ1a catalytic triad cysteine (C216) is essential for all described virulence and avirulence functions, as well as for its biochemical activities, and a HopZ1a^C216A^ mutant behaves as a catalytically inactive mutant (Ma et al., 2006; Lewis et al., 2008; Macho et al., 2010; Lee et al., 2012). *Xanthomonas* AvrBsT, the only other effector of the YopJ/HopZ superfamily with ETI-suppressing activity, shares with HopZ1a the biochemical activities, cofactor requirements, and catalytic triad dependence on its virulence and avirulence functions (Szczesny et al., 2010; Kim et al., 2013; Cheong et al., 2014). HopZ1a has also been shown to autoacetylate in a lysine residue (K289) conserved in some related effectors, with the HopZ1a^K289R^ mutant phenocopying the catalytically inactive HopZ1a^C216A^ mutant in respect to the acetyltransferase activity, and also to its avirulence and some of its virulence functions (Lee et al., 2012). Autoacetylation of such conserved lysine residue was originally described for another member of the YopJ/HopZ superfamily, *Ralstonia* effector PopP2 (Tasset et al., 2010). Auto-acetylation of PopP2 is required to trigger a defense response mediated by RRS1-R, a plant resistance protein that interacts with PopP2 but is not acetylated by this effector (Tasset et al., 2010).

In this work, we analyze the requirement of HopZ1a K289 acetylation for HopZ1a suppression of ETI and SAR, as well as its avirulence function, i.e., HopZ1a induction of ETI. We have found that expression of HopZ1a^K289R^ suppresses accumulation of PR1 in local tissue, as well as SAR in distal tissues, although the suppression activities of the mutant effector are not as efficient as those achieved by expression of wild type HopZ1a. Our results indicate that auto-acetylation of this residue is important for full activity but not essential for suppression of either ETI or SAR. Interestingly, we also found that the K289R mutation does not abolish the onset of ETI upon HopZ1a recognition, although it is required for full immunity. The K289R mutation reduces but does not prevent HopZ1a-mediated immunity from restricting growth of DC3000, in contrast with mutation of the C216 catalytic residue. Similarly, the K289R mutation reduces but does not abolish HopZ1a induction of macroscopic HR, and more importantly, it does not eliminate HopZ1a ability to effectively protect *Arabidopsis* against infection with DC3000. Our results indicate that this residue is important but not essential for HopZ1a activity, since its mutation does not abrogate the effector virulence and avirulence activities.

## Materials and Methods

### Bacterial Strains and Growth Conditions

*Pseudomonas syringae* pv. *tomato* DC3000 (Cuppels, 1986) and derivatives carrying a plasmid (**Table 1**), as well as *Agrobacterium tumefaciens* C58C1 (Deblaere et al., 1985), were grown at 28°C in Luria-Bertani (LB) medium. Antibiotics were used at the following concentration: 10 μg/ml gentamicin and 15 μg/ml kanamycin for *P. syringae* strains; 50 μg/ml kanamycin, 50 μg/ml rifampicin and 5 μg/ml tetracycline for *Agrobacterium.* All plates used to grow plant-extracted bacteria contained cycloheximide (2 μg/ml) to prevent fungal contamination.

**Table 1:** Plasmids used in this work.

Name	Promoter	Expressed effectors	Resistance	Reference
pAME30	*nptII*	HopZ1a	Amp, Km	Macho et al. (2010)
pAME27	*nptII*	HopZ1a^C216A^	Amp, Km	Macho et al. (2010)
pMAM1	*nptII*	HopZ1a^K289R^	Amp, Km	This work
pAME8	*nptII*	AvrRpt2	Amp, Km	Macho et al. (2009)
pAME33	*nptII*	HopZ1a + AvrRpt2	Amp, Km	Macho et al. (2010)
pAME34	*nptII*	HopZ1a^C216A^ + AvrRpt2	Amp, Km	Macho et al. (2010)
pJRU10	*nptII*	HopZ1a^K289R^ + AvrRpt2	Amp, Km	This work
pAME30Gm	*nptII*	HopZ1a	Amp, Km, Gm	This work
pAME27Gm	*nptII*	HopZ1a^C216A^	Amp, Km, Gm	This work
pMAM1Gm	*nptII*	HopZ1a^K289R^	Amp, Km, Gm	This work
pBINZ1	35S	6xHis-HopZ1a	Km	This work
pBINZ2	35S	6xHis-HopZ1a^C216A^	Km	This work
pBINZ3	35S	6xHis-HopZ1a^K289R^	Km	This work

### Plant Material and Bacterial Inoculations

*Arabidopsis thaliana* (Col-0) and the T-DNA insertion line *zar1-1* (Lewis et al., 2010) were grown in soil, or for disease symptom development assays, in jiffy-7 (Jiffy Products Ltd., Norway). In either case, they were grown in temperature-controlled chambers, at 21°C with a controlled photoperiod of 8 h light/16 h dark with a light intensity of 200 μmol/m^2^/s. *Nicotiana benthamiana* was grown in soil in temperature-controlled chambers, at 21°C with a controlled photoperiod of 16 h light/8 h dark with a light intensity of 200 μmol/m^2^/s.

Competitive index and canceled-out assays were performed as previously described for *Arabidopsis* (Macho et al., 2007). Using a blunt syringe, 4- to 5-weeks-old plants were inoculated with a 5 × 10^4^ cfu (colony-forming unit)/ml mixed bacterial suspension, containing equal numbers of wild type and effector-expressing strains. Serial dilutions of the inoculum were plated onto LB agar and LB agar with kanamycin to confirm dose and relative proportion between the strains, which should be close to one. We have previously established that two strains co-inoculated in an equaled-number inoculum at a 5 × 10^4^ cfu/ml concentration, grow as they would when inoculated individually (i.e., without any interference such as complementation or dominant negative effects on the growth of each other). Thus, by analyzing their growth within the same plant, we can carry out an accurate and direct comparison between their respective growths, by reducing plant-to plant and experimental deviations. At 2 or 4 days post-inoculation (dpi), three 10-mm-diameter leaf disks were homogenized into 1 ml of 10 mM MgCl_2_, by mechanical disruption. Bacteria were enumerated by plating serial dilutions onto LB agar with cycloheximide, and LB agar with kanamycin and cycloheximide, to differentiate the strains within the mixed infection. Bacterial enumeration was carried out in the dilution displaying between 50 and 500 colonies per plate. The CI is defined as the mutant-to-wild type ratio within the output sample divided by the mutant-to-wild type ratio within the input (inoculum; Freter et al., 1981; Taylor et al., 1987). The canceled-out index (COI) is calculated dividing the output ratio between the strain expressing two effectors and the strain expressing one effector, by their input ratio (Macho et al., 2010). Competitive and canceled-out indices presented are the mean of three biological replicates from at least three independent experiments (i.e., at least nine biological replicates). Errors bars represent standard error. Each CI or COI was analyzed using a homoscedastic and two-tailed Student’s *t*-test and the null hypothesis: mean index is not significantly different from one, or from other mean value (*P*-value < 0.05).

For measuring SAR, plants were initially inoculated with either 10 mM MgCl_2_ (mock), DC3000 or DC3000-expressing effectors at 5 × 10^5^ cfu/ml. After 2 dpi, secondary leaves were inoculated with a 5 × 10^4^ cfu/ml DC3000 suspension. Growth of DC3000 was measured in secondary leaves at 4 dpi, as already described.

For macroscopic HR assays, fully expanded leaves of 4- to 5-weeks-old *Arabidopsis* plants were inoculated using a blunt syringe with a 5 × 10^7^ cfu/ml bacterial suspension, and symptoms were documented at 20 or 24 h post-inoculation (hpi). A minimum of 30 leaves was inoculated per strain and plant genotype.

For transient expression assays in *N. benthamiana*, 5-weeks-old plants were inoculated with an *A. tumefaciens* C58C1 solution at OD_600_ 0.5 in 10 mM MgCl_2_, 10 mM MES (SIGMA, USA), 200 μM 3′,5′-dimethoxy-4′-hydroxyacetophenone (acetosyringone; SIGMA, USA) carrying the corresponding binary plasmids (**Table 1**). Plants were monitored for development of macroscopic HR and photographed at 48 h post-inoculation.

For protein extractions, two fully expanded *Arabidopsis* young leaves were inoculated with either 10 mM MgCl_2_ (mock) or a 5 × 10^5^ cfu/ml bacterial solution.

For symptom visualization, 3-weeks-old *Arabidopsis* plants were sprayed with a bacterial suspension containing 5 × 10^7^ cfu/ml in 10 mM MgCl_2_ containing 0.02% Silwet-L77 (Crompton Europe Ltd., UK). Plants were kept covered for 24 h to keep humidity high.

### Plasmid Generation

Plasmids used in this work are listed in **Table 1**. Parental vectors and cloning intermediaries are described in this section.

HopZ1a K289R point mutation was generated following the instructions for the QuikChange Lightning Multi Site-Directed Mutagenesis Kit (Agilent Technologies, USA) using vectors pAME30 and pAME33 as templates, to generate pMAM1 and pJRU10, respectively. The primers used were Z1aM1 (CCGGTGGATTTTTATAGGCATGGCGCTTCGCTG) and Z1aM2 (CAGCGAAGCGCCATGCCTATAAAAATCCACCGG). The point mutation was verified by sequencing.

For COI assays, a fragment containing a gentamicin resistance cassette was excised from pMGm (Murillo et al., 1994) using *Kpn*I and cloned into the corresponding site of pAME30 (HopZ1a), pAME27 (HopZ1a^C216A^), and pMAM1 (HopZ1a^K289R^), to generate pAME30Gm, pAME27Gm, and pMAM1Gm, respectively. The gentamicin resistance cassette allowed antibiotic selection of strains carrying these plasmids versus strains carrying plasmids conferring kanamycin resistance.

Vectors used for *in planta* transient expression assays were generated by means of an intermediate cloning step using expression vector pET28a(+) (Novagen, USA). HopZ1a, HopZ1a^C216A^ and HopZ1a^K289R^ were amplified by PCR with the iProof High-Fidelity PCR Kit (BioRad, USA), using plasmids pAME30, pAME27, and pMAM1 as templates, and primers Z1pET-F (AACATATGGGAAATGTATGCGTCG) and Z1pET-R (AAGGATCCTTAGCGCTGCTCTTCGGC). PCR-amplified DNA fragments, encoding the corresponding ORFs were digested with *Nde*I and *BamH*I and cloned into the corresponding sites of expression vector pET28a(+). The resulting vectors pET28-Z1a, pET28-C2, and pET28-K2 carry HopZ1a, HopZ1a^C216A^, and HopZ1a^K289R^ as 6xHis N-terminal fusion proteins, respectively. The ORFs for 6xHis-HopZ1a, 6xHis-HopZ1a^C216A^, and 6xHis-HopZ1a^K289R^ were excised from pET28-Z1a, pET28-C2, and pET28-K2 using *Xba*I and *BamH*I, and cloned into the corresponding sites of binary vector pBINX1 (Sanchez-Duran et al., 2011): the resulting vectors were designated pBINZ1, pBINZ2, and pBINZ3, respectively.

### Plant Protein Extraction and Western Blot

Approximately 100 μg of leaf tissue were harvested, frozen into liquid nitrogen and ground into 100 μl of extraction buffer [10 mM Tris-HCl pH 7.4, 150 mM NaCl and EDTA-free plant protease inhibitor cocktail (Roche, Germany)]. The resulting homogenate was centrifuged at 20000 *g* for 10 min at 4°C. Soluble supernatant was separated and centrifuged again to ensure absence of insoluble debris. Protein concentration of the soluble supernatant was determined by the BioRad protein assay (BioRad, USA). Ten micrograms of each protein sample, unless otherwise stated, were resolved on 12% acrylamide SDS-PAGE gels (Mini protean, BioRad, USA) and transferred to PVDF membranes (Millipore, USA). Western blots for immunodetection of PR1 were carried out using standard methods, with a 1:5000 dilution of anti-PR1 antibody and 1:10000 dilution of a secondary Anti-Rabbit antibody (SIGMA, USA). Membranes were developed using the BioRad Clarity Western ECL Substrate (BioRad, USA) following instructions from the manufacturer. The anti-PR1 serum was originally described by Wang et al. (2005).

## Results

### HopZ1a^K289R^ Suppresses Local PR1 Accumulation Triggered by DC3000

We have previously shown that HopZ1a suppresses DC3000-triggered PR1 protein accumulation, and that this suppression requires its catalytic cysteine C216 residue (Macho et al., 2010). To analyze the potential effect of the K289R mutation on HopZ1a activity, we inoculated *Arabidopsis* Col-0 plants with DC3000, DC3000 expressing HopZ1a, or DC3000 expressing either the catalytically inactive HopZ1a^C216A^ mutant or the HopZ1a^K289R^ mutant, and compared the levels of PR1 accumulation in local tissue 48 h after infection (hpi).

**FIGURE 1:**
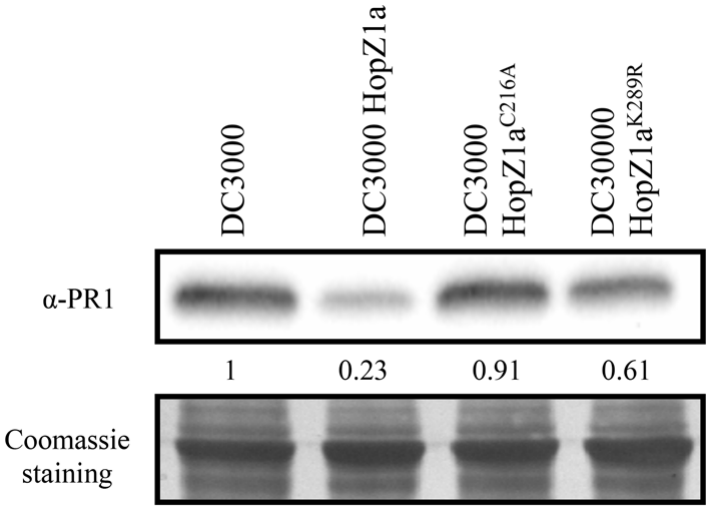
**HopZ1a-mediated suppression of local DC3000-triggered PR1 accumulation is reduced but not abolished by the K289R mutation.** Western blot showing PR1 accumulation in Col-0 leaves inoculated with 5 × 10^5^ cfu/ml of DC3000, DC3000 expressing HopZ1a (pAME30), or DC3000 expressing the mutant derivatives HopZ1a^C216A^ (pAME27) or HopZ1a^K289R^ (pMAM1). Ten micrograms of total protein were loaded per sample, and Coomassie staining is shown as loading control. The signal intensity for each band was quantified using Fiji distribution of ImageJ software and is shown below the blot. The experiment was repeated three times with similar results.

In keeping with our previous results (Macho et al., 2010), PR1 accumulated to similar levels in leaves inoculated with DC3000 or DC3000 expressing HopZ1a^C216A^, while PR1 accumulation was clearly reduced in plants inoculated with DC3000 expressing HopZ1a (**Figure 1**). When leaves were inoculated with DC3000 expressing the HopZ1a^K289R^ mutant protein, PR1 levels were slightly reduced compared to those observed following inoculation with DC3000 or DC3000 expressing HopZ1a^C216A^, however, this reduction was not as substantial as that achieved by the wild type version of the effector (**Figure 1**). These results indicate that the HopZ1a^K289R^ mutant is still able to suppress local PR1 accumulation elicited by virulent bacteria in the context of a compatible interaction, and suggest that the K289R mutation does not render the effector inactive, in contrast to the C216A mutation.

### HopZ1a^K289R^ Suppresses AvrRp2-Triggered Immunity

We have also described previously that HopZ1a suppresses the local accumulation of PR1 that accompanies the onset of the ETI triggered by the expression of the heterologous effector AvrRpt2 by DC3000 (Macho et al., 2010). HopZ1a suppression of AvrRpt2-induced PR1 accumulation is a virulence activity that also depends on the HopZ1a catalytic cysteine C216 (Macho et al., 2010). To analyze the potential effect of the K289R mutation on HopZ1a activity, we inoculated *Arabidopsis* Col-0 plants with DC3000 expressing AvrRpt2, or DC3000 co-expressing AvrRpt2 and either HopZ1a, HopZ1a^C216A^, or HopZ1a^K289R^, and compared the levels of PR1 in the inoculated tissue at 24 hpi.

**FIGURE 2:**
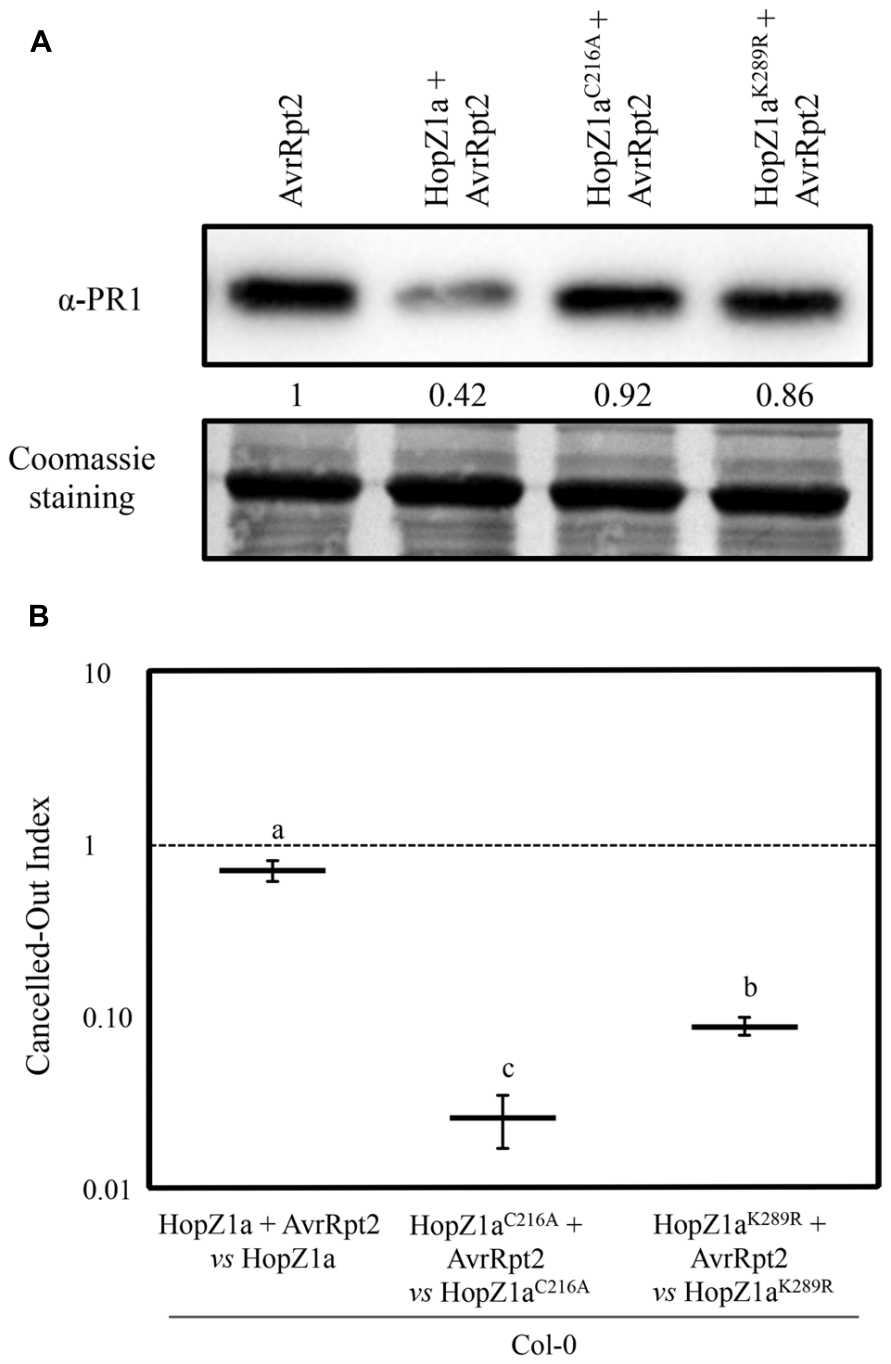
**HopZ1a^K289R^ partially suppresses AvrRpt2-triggered immunity. (A)** Western blot showing PR1 accumulation in Col-0 leaves inoculated with 5 × 10^5^ cfu/ml of DC3000 expressing AvrRpt2 (pAME8) alone or co-expressing AvrRpt2 with HopZ1a (pAME33), HopZ1a^C216A^ (pAME34), or HopZ1a^K289R^ (pJRU10). Ten micrograms of total protein were loaded per sample, and Coomassie staining is shown as loading control. The signal intensity for each band was quantified using Fiji distribution of ImageJ software and is shown below the blot. The experiment was repeated twice with similar results. **(B)** Canceled-out indices (COIs) measuring growth within a mixed infection of DC3000 co-expressing AvrRpt2 and any of the three HopZ1a variants: wild-type HopZ1a (pAME33), HopZ1a^C216A^ (pAME34) or HopZ1a^K289R^ (pJRU10), in relation to growth of DC3000 expressing only the corresponding HopZ1a: wild-type HopZ1a (pAME30Gm), HopZ1a^C216A^ (pAME27Gm), or HopZ1a^K289R^ (pMAM1Gm). COIs are calculated as the output ratio between the strain expressing both effectors and the strain expressing just one, divided by their input ratio. Each COI value represents the means of two independent experiments with three biological replicates each. Error bars represent the standard error. Mean values marked with the same letter are not significantly different from each other as established by Student’s *t*-test (*P* < 0.05).

PR1 accumulated to similar levels in leaves inoculated with DC3000 expressing AvrRpt2 or DC3000 co-expressing AvrRpt2 and HopZ1a^C216A^, while PR1 accumulation was clearly reduced in leaves inoculated with DC3000 co-expressing AvrRpt2 and HopZ1a (**Figure 2A**). In leaves inoculated with DC3000 co-expressing AvrRpt2 and the HopZ1a^K289R^ mutant protein, we could not detect differences in PR1 accumulation in comparison to leaves inoculated with either DC3000 expressing AvrRpt2 alone or with HopZ1a^C216A^ (**Figure 2A**)

HopZ1a suppression of AvrRpt2-triggered defense responses has also been demonstrated in *Arabidopsis* by directly comparing the growth attenuation determined by the individual expression of each effector, with the growth attenuation determined by their simultaneous expression (Macho et al., 2010). Thus, we also analyzed the impact of the K289R mutation on the suppression of AvrRpt2-triggered growth restriction. To do so we performed mixed infections and calculated the COI, a modification of the competitive index (Beuzón and Holden, 2001), previously applied to this purpose (Macho et al., 2010). COIs directly measure the differences in growth, within the same plant, between a strain expressing one of the effectors and a strain co-expressing both effectors, i.e., differences in growth of co-inoculated DC3000 expressing HopZ1a and DC3000 co-expressing HopZ1a and AvrRpt2. Thus, we can directly compare how expression of AvrRpt2 affects growth of DC3000 in the presence of HopZ1a or any of its mutant derivatives, with growth of DC3000 expressing only the HopZ1a version. As HopZ1a is expressed in both strains, the growth reduction it causes in Col-0 is canceled out as it equally affects both strains (Macho et al., 2010), and any difference in growth detected between the strain expressing both effectors and the strain expressing only the HopZ1a version, would be due to a growth restriction determined by the unsuppressed defenses triggered against AvrRpt2. A diagram illustrating this analysis is included as supplementary material (Supplementary Figure S1).

As previously reported (Macho et al., 2009, 2010) DC3000 co-expressing AvrRpt2 and HopZ1a displayed a small although significant growth attenuation compared to that of co-inoculated DC3000 only expressing HopZ1a (COI = 0.69 ± 0.09; **Figure 2B**, Supplementary Figure S1), despite the fact that AvrRpt2 alone triggers a 50–100 fold growth attenuation when expressed by DC3000 from the same plasmid. This result is expected since HopZ1a is capable of partially suppressing the defense response triggered by AvrRpt2 in *Arabidopsis* (Macho et al., 2010). Accordingly, growth of DC3000 co-expressing AvrRpt2 and the HopZ1a^C216A^ catalytic mutant was almost 50 fold lower than the growth of DC3000 expressing HopZ1a^C216A^ alone (COI = 0.03 ± 0.01; **Figure 2B**, Supplementary Figure S1). However, growth of DC3000 co-expressing AvrRpt2 and the HopZ1a^K289R^ mutant was only a 10-fold lower than growth of the strain expressing HopZ1a^K289R^ alone (COI = 0.09 ± 0.01). These results indicate that mutation K289R decreases, but does not abrogate, HopZ1a ability to suppress AvrRpt2-triggered restriction of growth, since co-expression of AvrRpt2 and HopZ1a^K289R^ causes a smaller attenuation of growth than co-expression of AvrRpt2 and the HopZ1a^C216A^ catalytic mutant or expression of AvrRpt2 alone (**Figure 2B**).

Our results (**Figure 2B**) indicate that, unlike the catalytically inactive HopZ1a^C216A^ mutant derivative, HopZ1a^K289R^ mutant is still able to suppress AvrRpt2-triggered immunity, since it still suppresses AvrRpt2-triggered restriction of growth. The fact that we do not detect suppression of PR1 protein in plants inoculated with DC3000 expressing the HopZ1a^K289R^ mutant may indicate that our assay is not sensitive enough, or that the association between the PR1 accumulation and growth restriction associated to AvrRpt2-triggered immunity is not linear. To this regards, a similar lack of linearity in the association between PR1 accumulation and growth restriction during induction of SAR has been previously shown (Macho et al., 2010).

### HopZ1a Partially Suppresses AvrRpt2-Triggered Immunity in *zar1-1* Mutant Plants

Results presented in **Figure 2B** are in agreement with our previous report concluding that HopZ1a partially suppresses AvrRpt2-triggered ETI in *Arabidopsis* (Macho et al., 2010). However, it has been recently reported that HopZ1a transgenic expression in *zar1-1* plants does not interfere with AvrRpt2-induced macroscopic HR (Lewis et al., 2014). HopZ1a-triggered immunity in *Arabidopsis* is dependent on the ZAR1 resistance protein (Lewis et al., 2010). In the light of this report we decided to analyze the ability of HopZ1a to suppress AvrRpt2-triggered immunity in the absence of HopZ1a-triggered immunity. To this purpose, we analyzed HopZ1a impact on AvrRpt2-triggered restriction of growth in a *zar1-1* plant genotype (Lewis et al., 2010).

**FIGURE 3:**
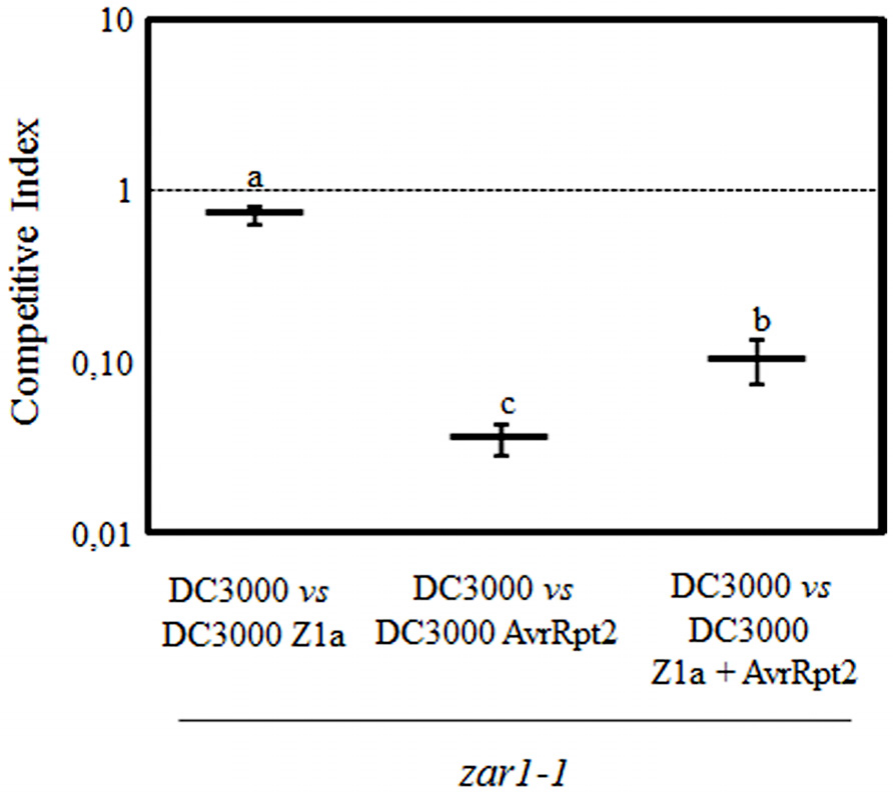
**HopZ1a suppresses AvrRpt2-triggered ETI in *zar1-1* plants.** Competitive indices (CIs) measuring growth within a mixed infection of DC3000 expressing HopZ1a (Z1a, pAME30), AvrRpt2 (pAME8) or co-expressing both (Z1a + AvrRpt2, pAME33) in relation to growth of DC3000. CIs are calculated as the output ratio between the strain expressing the effector(s) and DC3000, divided by their input ratio. Each CI value represents the means of three independent experiments with three biological replicates each. Error bars represent the standard error. Mean values marked with the same letter were not significantly different from each other as established by Student’s *t*-test (*P* < 0.05).

Using CI assays, we compared growth of DC3000 expressing HopZ1a or AvrRpt2 with growth of DC3000 in *zar1-1* plants, to determine the growth restriction associated to ETI responses against each of these effectors in the mutant background (**Figure 3**). Growth of DC3000 expressing HopZ1a was very similar to growth of DC3000 in *zar1-1* plants (CI = 0.72 ± 0.09; **Figure 3**). Whereas, as expected since AvrRtp2-triggered immunity is independent of ZAR1, the expression of this effector in DC3000 still determined a strong growth attenuation (CI = 0.03 ± 0.01; **Figure 3**). However, co-expression of AvrRpt2 and HopZ1a in *zar1-1* plants caused significantly less growth attenuation (CI = 0.10 ± 0.03) than that caused by expression of AvrRpt2 alone (**Figure 3**), demonstrating that HopZ1a suppression of AvrRpt2-triggered immunity takes place in the absence of HopZ1a-triggered immunity, and is not caused by an overlap or interference between the two ETI pathways.

### HopZ1a^K289R^ Retains the Ability to Suppress Systemic Acquired Resistance (SAR) Triggered by DC3000 Infection

**FIGURE 4:**
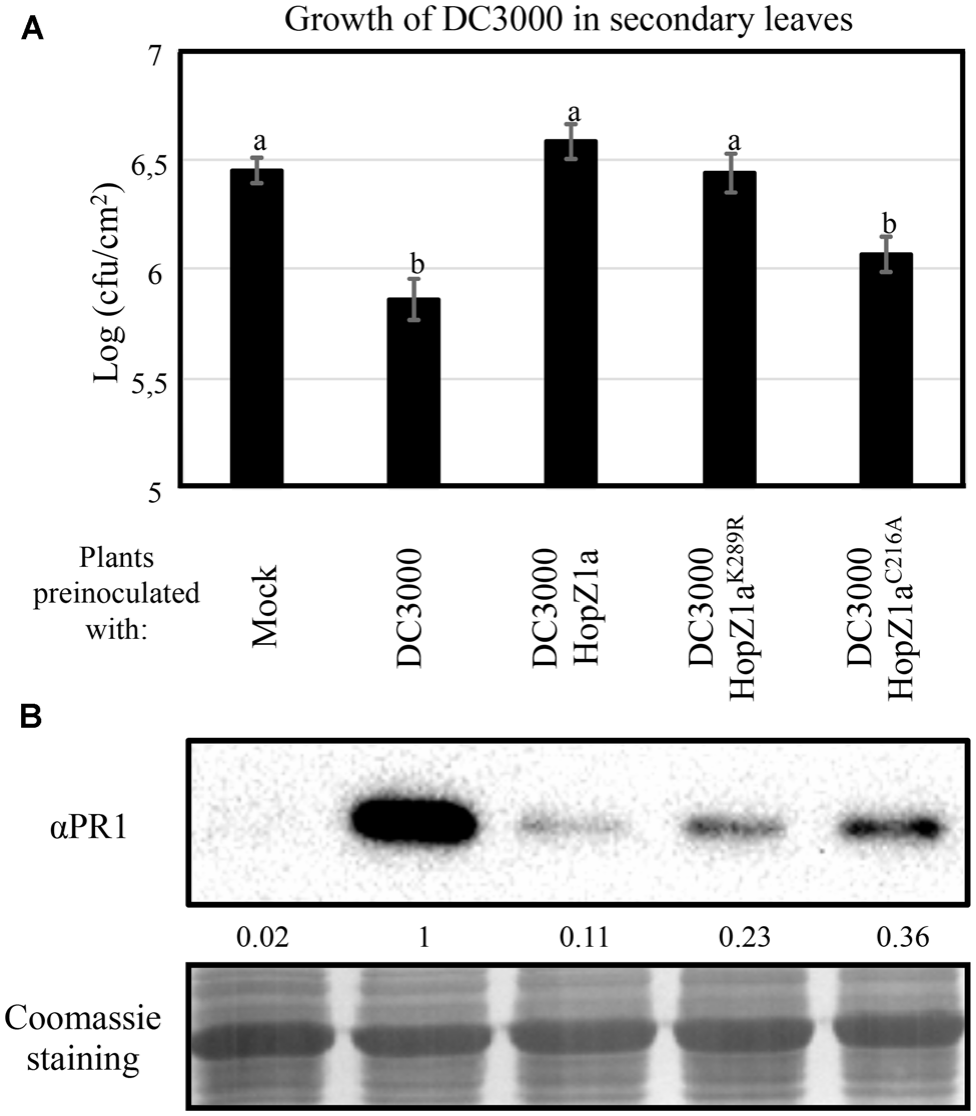
**HopZ1a-mediated suppression of SAR is reduced but not abolished by the K289R mutation. (A)** Growth of DC3000 inoculated in secondary leaves of plants pre-inoculated in primary leaves by infiltrating either a 10 mM MgCl_2_ solution (Mock), or 5 × 10^5^ cfu/ml of DC3000, DC3000 expressing HopZ1a (pAME30), or DC3000 expressing the mutant derivatives HopZ1a^C216A^ (pAME27) or HopZ1a^K289R^ (pMAM1). Two days post-inoculation of primary leaves, secondary leaves were inoculated with 5 × 10^4^ cfu/ml of DC3000, and growth was measured at 4 days post-inoculation of the secondary leaves. The experiment was repeated four times with similar results, and the results shown correspond to a representative experiment. The values shown represent the means of 5 biological replicates. Error bars represent the standard error. Values marked with the same letter were not significantly different from each other as established by Student’s *t*-test (*P* < 0.05). **(B)** Western blot analysis for immunodetection of PR1 on distal non-inoculated leaves, 2 days after inoculating primary leaves with either 10 mM MgCl_2_ (Mock), or 5 × 10^5^ cfu/ml of DC3000, DC3000 expressing HopZ1a (pAME30), or DC3000 expressing the mutant derivatives HopZ1a^C216A^ (pAME27) or HopZ1a^K289R^ (pMAM1). Ten micrograms of total protein were loaded per sample, and Coomassie staining is shown as loading control. The signal intensity for each band was quantified using Fiji distribution of ImageJ software and is shown below the blot. The experiment was repeated twice with similar results.

Both virulent and avirulent bacteria can trigger SAR, a defense response elicited in distal (systemic) tissues as a result of local infection. Activation of SAR determines both systemic accumulation of PR1, and restriction of growth of newly incoming bacteria (Cameron et al., 1994). We have previously shown that HopZ1a expression suppresses SAR triggered by DC3000, and that such suppression requires HopZ1a catalytic cysteine C216 (Macho et al., 2010). To determine whether the HopZ1a^K289R^ mutant retained HopZ1a ability to suppress SAR, we first analyzed the effect of the mutation K289R in HopZ1a ability to suppress SAR-associated restriction of growth of newly incoming bacteria. We inoculated primary leaves with either 10 mM MgCl_2_ (mock), DC3000, or DC3000 expressing HopZ1a or the corresponding mutant derivatives HopZ1a^C216A^ or HopZ1a^K289R^ (**Figure 4A**). Two days after inoculation of primary leaves, distal leaves were inoculated with DC3000, and 4 days after this second inoculation we monitored the growth of DC3000. **Figure 4A** shows that, as previously described, pre-inoculation of primary leaves with either DC3000 or DC3000 expressing the catalytically inactive mutant HopZ1a^C216A^ triggered SAR to equivalent levels, since growth of DC3000 in distal leaves was similarly attenuated in both cases. In contrast, pre-inoculation with DC3000 expressing HopZ1a did not result in detectable attenuation of growth of DC3000 in distal leaves, since it did not show significant differences with that observed in mock pre-inoculated leaves, thus confirming the reported HopZ1a suppression of SAR (Macho et al., 2010). Systemic leaves from plants pre-inoculated with DC3000 expressing HopZ1a^K289R^ displayed DC3000 cfu values significantly different to those reached in plants pre-inoculated with DC3000 expressing HopZ1a^C216A^, but similar to those reached in plants pre-inoculated with DC3000 expressing HopZ1a (**Figure 4A**), supporting the notion that auto-acetylation of HopZ1a on K289 is not required for suppression of SAR in *Arabidopsis*.

To determine how the HopZ1a^K289R^ mutant ability to suppress SAR correlates with suppression of PR1 accumulation in systemic tissue, we used western blot analysis to analyze accumulation of PR1 in systemic leaves of plants pre-inoculated with DC3000 or DC3000 expressing the different versions of HopZ1a. In keeping with previous results (Macho et al., 2010), expression of HopZ1a in DC3000 suppresses PR1 accumulation in systemic tissues, since distal leaves of plants pre-inoculated with DC3000 expressing HopZ1a displayed a strong reduction of PR1 accumulation when compared to plants pre-inoculated with DC3000 (**Figure 4B**). This suppression is dependent on HopZ1a catalytic cysteine, since systemic leaves of plants pre-inoculated with DC3000 expressing HopZ1a^C216A^ displayed PR1 levels that were higher than those observed in plants pre-inoculated with DC3000 expressing HopZ1a (**Figure 4B**). As previously reported (Macho et al., 2010) the C216A mutation did not entirely abolish HopZ1a ability to suppress PR1 accumulation, since the systemic levels of PR1 in plants pre-inoculated with DC3000 expressing HopZ1a^C216A^ did not reach the levels observed in plants pre-inoculated with DC3000 (**Figure 4B**). Interestingly, when primary leaves were inoculated with DC3000 expressing HopZ1a^K289R^, the accumulation of PR1 in distal leaves reached levels that were intermediate between those elicited by DC3000 expressing HopZ1a^C216A^ and DC3000 expressing wild type version of the effector (**Figure 4B**). These results indicate that the HopZ1a^K289R^ mutant is still able to partially suppress systemic accumulation of PR1 in response to DC3000.

### HopZ1a^K289R^ Triggers ETI in *Arabidopsis* and *N. benthamiana*

Inoculation of *Arabidposis* leaves with a 5 × 10^7^ cfu/ml of DC3000 expressing HopZ1a induces macroscopic HR symptoms in *Arabidopsis* leaves, which are absent in leaves inoculated with the same dose of DC3000 expressing the HopZ1a^C216A^ catalytic mutant (Lewis et al., 2008; Macho et al., 2010). It has been reported that the mutation K289R completely prevents HopZ1a-triggered HR, which cannot be detected when expressing the mutant effector under the control of its own promoter (Lee et al., 2012). However, considering that our results presented above indicate that such mutation does not render the effector entirely inactive, we wondered whether HopZ1a^K289R^ could still be able to trigger immunity in *Arabidopsis*.

**FIGURE 5:**
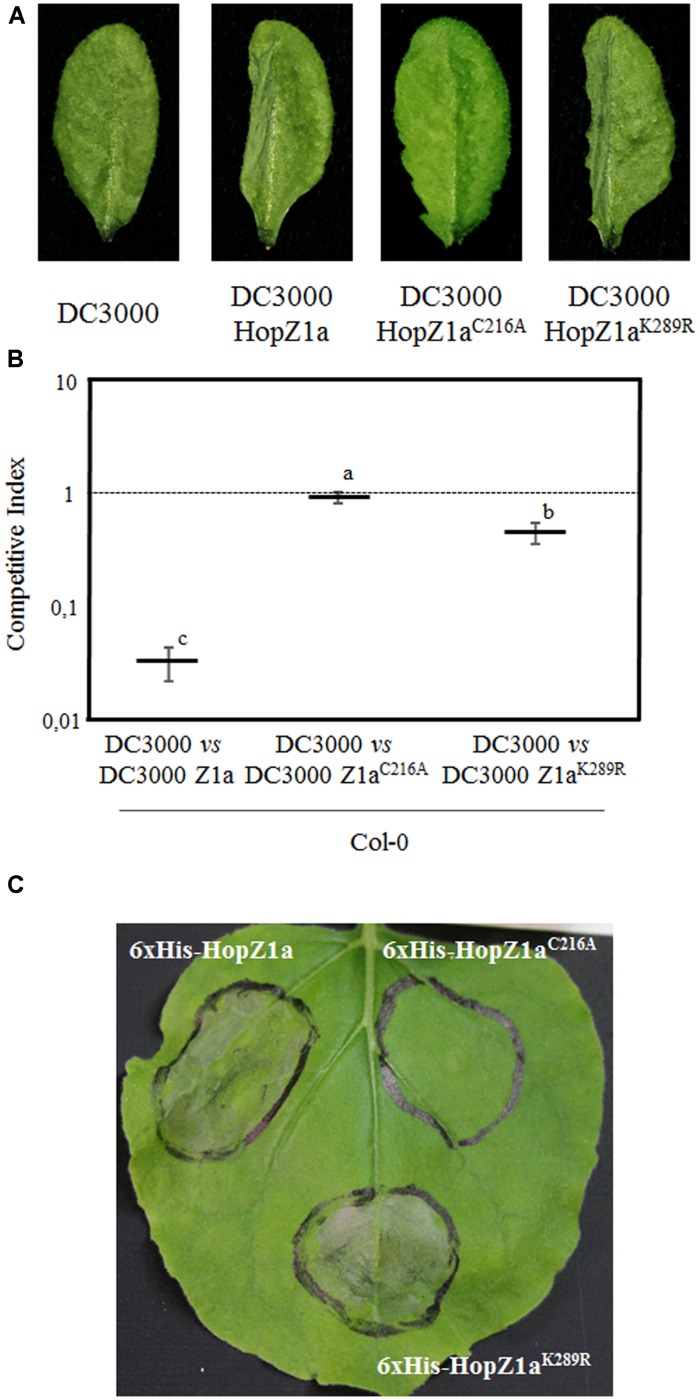
**HopZ1a^K289R^ triggers ETI. (A)** Hypersensitive response (HR) to hand-infiltration of Col-0 leaves with bacterial suspensions containing 5 × 10^7^ cfu/ml of DC3000 alone or DC3000 expressing HopZ1a (pAME30), or each of its mutant derivatives HopZ1a^C216A^ (pAME27) or HopZ1a^K289R^ (pMAM1). Photographs were taken 24 h post-inoculation. Images are representative of at least 30 inoculated leaves per strain and experiment. The experiment was repeated twice with similar results. **(B)** Competitive indices (CIs) measuring growth within a mixed infection of DC3000 expressing HopZ1a (Z1a, pAME30), or each of its mutant derivatives HopZ1a^C216A^ (Z1a^C216A^, pAME27) or HopZ1a^K289R^ (Z1a^K289R^, pMAM1), in relation to growth of DC3000. CIs are calculated as the output ratio between the strain expressing the effector and DC3000, divided by their input ratio. Each CI value represents the means of three independent experiments with three biological replicates each. Error bars represent the standard error. Mean values marked with the same letter were not significantly different from each other as established by Student’s *t*-test (*P* < 0.05). **(C)** Development of HR following transient expression of either 6xHis-HopZ1a (pBINZ1) or each of its mutant derivatives 6xHis-HopZ1a^C216A^ (pBINZ2) or 6xHis-HopZ1a^K289R^ (pBINZ3). *Nicotiana benthamiana* leaves were inoculated with *Agrobacterium tumefaciens* C58C1 carrying binary plasmids encoding the corresponding effector genes. Pictures were taken 48 h post-inoculation. The experiment was repeated three times with similar results.

To analyze whether the HopZ1a^K289R^ mutant was able to trigger macroscopic HR in *Arabidopsis*, we inoculated leaves with 5 × 10^7^ cfu/ml of DC3000, DC3000 expressing HopZ1a, or DC3000 expressing either HopZ1a^C216A^ or HopZ1a^K289R^ mutant derivatives, and monitored HR development by 20–24 hpi (**Figure 5A**). Development of macroscopic HR requires a rather strong ETI response, which might not be reached by lower levels of effector-expression (Macho et al., 2009), thus we expressed HopZ1a and its mutant derivatives under the control of the strong constitutive *nptII* promoter, to factor in the chance of an stronger effector-expression allowing detection of a mild ETI. In keeping with previous reports (Lewis et al., 2008; Macho et al., 2010), a clear HR was detected in plants inoculated with DC3000 expressing HopZ1a, while no HR could be detected in leaves inoculated with either DC3000 or DC3000 expressing HopZ1a^C216A^ (**Figure 5A**). Interestingly, leaves inoculated with DC3000 expressing HopZ1a^K289R^ displayed noticeable macroscopic HR (**Figure 5A**). As expected from previous reports (Lewis et al., 2010, 2014), inoculation of *zar1-1* leaves with these strains did not induce any visible cell death symptom (Supplementary Figure S2).

The ETI triggered in *Arabidopsis* against HopZ1a determines a strong restriction of bacterial growth that can be measured using competitive index assays (CIs), in mixed infections of DC3000 co-inoculated with DC3000 expressing HopZ1a (Macho et al., 2009, 2010). To further investigate the impact of the K289R mutation in HopZ1a activation of ETI in *Arabidopsis*, we performed CI assays by co-inoculating *Arabidopsis* plants with DC3000 and DC3000 expressing HopZ1a, HopZ1a^C216A^, or HopZ1a^K289R^ (**Figure 5B**). As previously described (Macho et al., 2010), a clear growth attenuation was measured for DC3000 expressing HopZ1a in comparison with co-inoculated DC3000 (CI = 0.03 ± 0.01), while no significant attenuation was detected for DC3000 expressing HopZ1a^C216A^ catalytically inactive (CI = 0.91 ± 0.10; **Figure 5B**). In contrast, DC3000 expressing HopZ1a^K289R^ displayed a small attenuation of growth (CI = 0.46 ± 0.10), significantly smaller than that measured for DC3000 expressing HopZ1a, but significantly different from the absence of attenuation observed for HopZ1a^C216A^-expressing DC3000 bacteria (**Figure 5B**).

In addition to triggering HR in *Arabidopsis*, HopZ1a has been shown to trigger macroscopic HR in *N. benthamiana* leaves when transiently expressed using *Agrobacterium* (Ma et al., 2006; Lewis et al., 2008). We expressed HopZ1a and its mutant derivatives HopZ1a^C216A^ and HopZ1a^K289R^ in *N. benthamiana* leaves, under the control of a constitutive promoter, by using *Agrobacterium*-mediated transient expression, and monitored HR symptoms at 40 h after *Agrobacterium* inoculation. While transient HopZ1a^C216A^ overexpression did not result in HR elicitation whatsoever, HopZ1a^K289R^ overexpression elicited an HR of a similar intensity to that elicited by overexpressing HopZ1a (**Figure 5C**).

Taken together, results shown in **Figure 5** indicate that auto-acetylation of HopZ1a in its lysine 289 contributes, but it is not essential, to trigger ETI in *Arabidopsis*.

### HopZ1a^K289R^-Triggered Defenses Effectively Protects *Arabidopsis* against Disease Development

**FIGURE 6:**
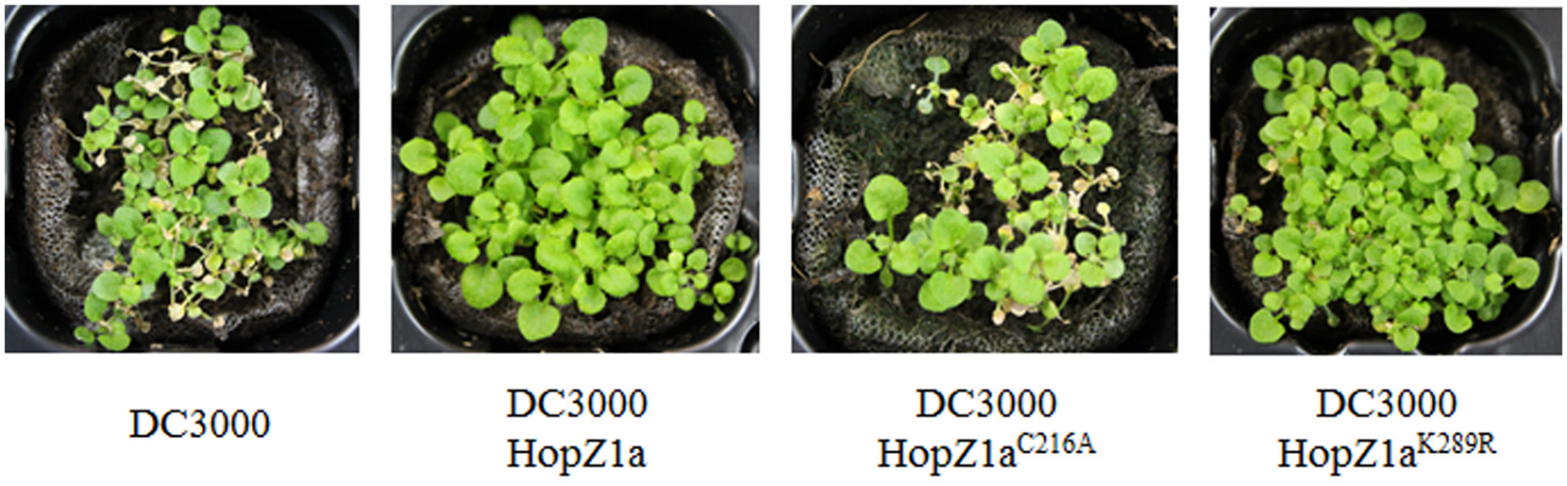
**Expression of HopZ1a^K289R^ from DC3000 protects Col-0 plants against disease.** Virulence symptoms caused by spray-inoculated DC3000, or DC3000 expressing HopZ1a (pAME30), or each of its mutant derivatives HopZ1a^C216A^ (pAME27) or HopZ1a^K289R^ (pMAM1). *Arabidopsis* plants were sprayed with bacterial suspensions containing 5 × 10^7^ cfu/ml in 0.02% Silwet L-77, and photographed 6 days post-inoculation. The experiment was repeated three times using five plants per strain, and representative images are shown.

We have previously shown that resistance triggered in *Arabidopsis* by the expression of HopZ1a efficiently protects plants from DC3000 infection, resulting in the absence of virulence-associated disease symptoms (Macho et al., 2010). To analyze whether the defense response triggered against HopZ1a^K289R^ mutant is sufficient to stymie DC3000 disease in *Arabidopsis*, we monitored development of disease symptoms at 4–6 dpi on plants spray-inoculated with DC3000, DC3000 expressing HopZ1a, or DC3000 expressing either HopZ1a^C216A^ or HopZ1a^K289R^ mutant derivatives. As expected from previous results (Macho et al., 2010), plants sprayed with either DC3000 or DC3000 expressing HopZ1a^C216A^ displayed noticeable disease symptoms, namely chlorosis and stunted growth, while plants sprayed with DC3000 expressing HopZ1a did not (**Figure 6**). Interestingly, plants sprayed with DC3000 expressing HopZ1a^K289R^ did not display chlorosis and only a slightly reduction in plant growth could be observed (**Figure 6**).

These results clearly show that the HopZ1a^K289R^ mutant triggered-resistance effectively protects *Arabidopsis* plants from disease.

## Discussion

### HopZ1a^K289R^ Triggers ETI in *Arabidopsis* and *N. benthamiana* and Effectively Protects *Arabidopsis* against Disease Development

Results described in this paper indicate that the HopZ1a^K289R^ mutant is still able to trigger a defense response that induces macroscopic HR (**Figure 5**) and, more importantly, effectively protects *Arabidopsis* from infection (**Figure 6**). While our results in respect to HopZ1a^K289R^ mutant triggering macroscopic HR are at variance with those described previously (Lee et al., 2012), this discrepancy can be due to differences in effector levels, since we express HopZ1a under the control of a strong constitutive promoter, and macroscopic HR symptoms can be quite dependent on threshold levels of the eliciting effectors (Macho et al., 2009). While the development of macroscopic HR symptoms after high-dose bacterial inoculation is a good measure of the ability of an effector to trigger a defense response, the protection from infection after a low-dose inoculation may reflect more accurately the physiological significance of such defense response for the plant. In this respect, results shown in **Figure 6** support the notion that the K289R mutation does not abrogate HopZ1a avirulence activity. It is important to notice that in neither of these assays, regarding induction of macroscopic HR or protection against disease development, did the catalytically inactive HopZ1a^C216A^ trigger any defense response whatsoever, regardless of being expressed under the control of the same strong promoter, supporting the notion that HopZ1a^K289R^ activity is not an artifact due to overexpression.

### HopZ1a^K289R^ Suppresses DC3000-Triggered Basal Immunity

Results described in this paper indicate that the HopZ1a^K289R^ mutant retains its ability to suppress basal immunity triggered by DC3000, since it can partially suppress DC3000-triggered local and systemic PR1 accumulation (**Figures 1** and **4**), as well as DC3000-triggered SAR-dependent restriction of growth in systemic tissues (**Figure 4**).

The fact that PR1 accumulation against DC3000 requires a functional T3SS (Macho et al., 2010) suggests that it is due to weak ETI-like defenses. However, the implication of PTI cannot be ruled out, since the level of PR1 accumulated in response to PAMPs in the attenuated T3SS mutant could be below the level of detection. In regards to this, a recent report demonstrates that HopZ1a can indeed suppress DC3000-triggered PTI response when overexpressed in transgenic *Arabidopsis* plants (Lewis et al., 2014). Additionally, the related effector AvrBsT can also suppress the PTI triggered by DC3000 infection in tomato, as shown by a lower accumulation of PR1 in the infected plants (Kim et al., 2010).

Previous reports have suggested that the K289R mutation completely abrogates HopZ1a virulence activity, since heterologous expression of the corresponding mutant effector in *P. syringae* pv. *cilantro* 0788-9 did not contribute to the growth of the expressing strain in the *Arabidopsis zar1-1* background (Lee et al., 2012). However, the rather modest growth rate achieved in *Arabidopsis* by *Pseudomonas* strain 0788-9, together with the limited contribution of wild type HopZ1a to such growth, might be limiting the sensitivity of such assay. Using a wider array of virulence assays, we demonstrate that the K289R mutation does not abolish HopZ1a virulence activity.

### HopZ1a Suppresses AvrRpt2-Triggered Immunity in the Absence of HopZ1a-Triggered Immunity

Our results demonstrate that HopZ1a suppression of AvrRpt2-triggered immunity takes place in the absence of HopZ1-triggered immunity (**Figure 3**), and it is therefore not a consequence of an overlap or interference between the defense responses triggered by these effectors. This notion was previously supported by the fact that AvrRpt2 does not alter HopZ1a-triggered restriction of growth in *rps2* plants, where AvrRpt2 does not trigger resistance but still displays virulence activity (Macho et al., 2010). This is in disagreement with a recent report based on transgenic overexpression of HopZ1a in *zar1-1* plants where it has been shown to suppress PTI, but not to prevent the onset of macroscopic HR (Lewis et al., 2014). However, such a strong suppression of PTI could alter potentially the ETI response of the transgenic lines.

### HopZ1a^K289R^ Suppresses AvrRpt2-Triggered ETI

Effector-triggered immunity suppression is a key virulence activity of HopZ1a that is only partly affected by the K289R mutation, while being completely eliminated in the catalytically inactive HopZ1a^C216A^ mutant. The related *Xanthomonas* effector AvrBsT has also been shown to suppress the ETI induced in pepper plants by a second *Xanthomonas* effector, AvrBs1 (Szczesny et al., 2010). In fact, the demonstrations of the ETI-suppressing activities of AvrBsT and HopZ1a were presented simultaneously, becoming the first known effectors belonging to the YopJ/HopZ family to display such virulence function (Macho et al., 2010; Szczesny et al., 2010). The ETI-suppression ability of AvrBsT is also dependent on its catalytic cysteine (Szczesny et al., 2010). However, the activity of the AvrBsT mutant equivalent to HopZ1a^K289R^ has only been assayed by heterologous expression in the interaction model based on *Pseudomonas* DC3000 and the *Arabidopsis* Pi-0 ecotype, where it does not display any virulence function (Cheong et al., 2014). In view of the new results presented here, and the similarities between HopZ1a and AvrBsT, it would be interesting to analyze the performance of the AvrBsT^K282R^ mutant in pepper plants, where it displays ETI-suppression abilities.

### ZAR1-Mediated Resistance against HopZ1a

HopZ1a has been reported in turn to modestly enhance the growth of DC3000 in *zar1-1* plants (Lewis et al., 2010) and to modestly decrease it (Jiang et al., 2013), in both cases differences in growth were statistically significant but within the same log. In our experimental conditions growth of DC3000 expressing HopZ1a was close to that of DC3000 in *zar1-1* plants, albeit slightly decreased (**Figure 3**), since the CI of DC3000 expressing HopZ1a in mixed infection with DC3000 (CI = 0.72 ± 0.09) was statistically different from 1.0. Our results are therefore in agreement with the observations of (Jiang et al., 2013). The disparity with the results from the first report (Lewis et al., 2010) could be due to differences in experimental settings, however, the existence of a residual defense against HopZ1a in the *Arabidopsis zar1-1* background cannot be ruled out. Such residual defense could be either due to residual activity of a truncated ZAR1 protein, or to a weak recognition by a second resistance protein, as described for other effectors (Saucet et al., 2015).

### HopZ1a Acetyltransferase Activity

On view of the results presented in this paper, the absolute requirement of the K289 lysine residue for HopZ1a activity can be discarded. In fact, it has recently been suggested in relation to the closely related effector HopZ1c (Lewis et al., 2014) that the C-terminal third of the HopZ family might be dispensable for acetyltransferase activity, or that HopZ1c may use water instead of acetyl-CoA during its enzymatic reaction, resulting in hydrolysis of substrates rather than acetylation, in an alternative catalytic mechanism suggested by Mukherjee et al. (2007). Furthermore, although the described autoacetylation site of HopZ1a is a lysine residue, the only HopZ1a interacting protein where the acetylated residues have been identified, namely the pseudokinase ZED1, is acetylated on threonine residues rather than lysines (Lewis et al., 2013). It seems therefore likely that HopZ1a might display acetyltransferase activity on amino acid residues, such as serine or threonine, as it is the case with YopJ, the archetypal effector of the YopJ/HopZ superfamily (Mukherjee et al., 2006).

Results presented to date for several YopJ/HopZ effectors do not support a consistent association between their autoacetylation at the conserved lysine residue and their transacetylation activities. For instance, AvrBsT maintains the autoacetylation activity in the absence of the conserved lysine residue, which is, however, essential for the acetylation of one of its described targets (Cheong et al., 2014). On the other hand, prior autoacetylation of YopJ is not required for acetylation of MEK, one of its described targets (Mittal et al., 2010). This opens the possibility that the said lysine residue and/or its autoacetylation, while contributing to the overall function of the effectors, is not essential for all their activities *in planta*. Considering the various targets proposed for each of the effectors belonging to the YopJ/HopZ superfamily, and the numerous biochemical activities assigned, sometimes concurrently, to said effectors (Orth et al., 2000; Hotson and Mudgett, 2004; Ma et al., 2006; Mukherjee et al., 2006; Sweet et al., 2007; Szczesny et al., 2010; Tasset et al., 2010; Zhou et al., 2011; Lee et al., 2012; Jiang et al., 2013; Lewis et al., 2013; Cheong et al., 2014), it is tempting to speculate that their molecular mechanisms *in planta* might be manifold, and therefore that the conserved lysine residue and/or its autoacetylation might not be required for all targets or activities. The resultant of all these activities on numerous targets would be observed as virulence or avirulence manifestations on a given plant model, and might be behind the intermediate phenotypes described for the HopZ1a^K289R^ mutant in this paper.

## Author Contributions

The conception and design of the work can be attributed to JR, AM, EB, CB, and JR-A. The acquisition of data and its primary analysis has been the responsibility of JR, AL, AM, BO-N, and MA-M, while ALL authors contributed to the final interpretation of the data. The paper has been drafted by the combined efforts of JR, AL, AM, BO-N, MA-M, CB, and JR-A, with the final version settled by JR, AM, EB, CB, and JR-A after critical revision. ALL authors approved the final version sent to the Editor of Frontiers in Microbiology for its review, and agree to be accountable for the accuracy and integrity of their respective contributions to the work presented in this paper.

## Conflict of Interest Statement

The authors declare that the research was conducted in the absence of any commercial or financial relationships that could be construed as a potential conflict of interest.

## Abbreviations

DC3000: *P. syringae* pathovar *tomato* strain DC3000
ETI: effector-triggered immunity
HR: hypersensitive response
PAMP: pathogen associated molecular patterns
PR1: pathogenesis related-1
PTI: PAMP-triggered immunity
T3SS: type III secretion system
