# Supplementary material for: Auto-acetylation on K289 is not essential for HopZ1a-mediated plant defense suppression
Source: Front Microbiol. 2015 Jul 8;6:684. doi: 10.3389/fmicb.2015.00684 (PMC4495678; doi:10.3389/fmicb.2015.00684)
Supplement: Supplementary file 1 [file Image_1.PDF]

A

$$\text{COI} = \frac{\text{DC3000 pZ1*+Rpt2 cfu}}{\text{DC3000 pZ1* cfu}}\ (\text{output}) \Big/ \frac{\text{DC3000 pZ1*+Rpt2 cfu}}{\text{DC3000 pZ1* cfu}}\ (\text{input})$$

* any version of HopZ1a

B

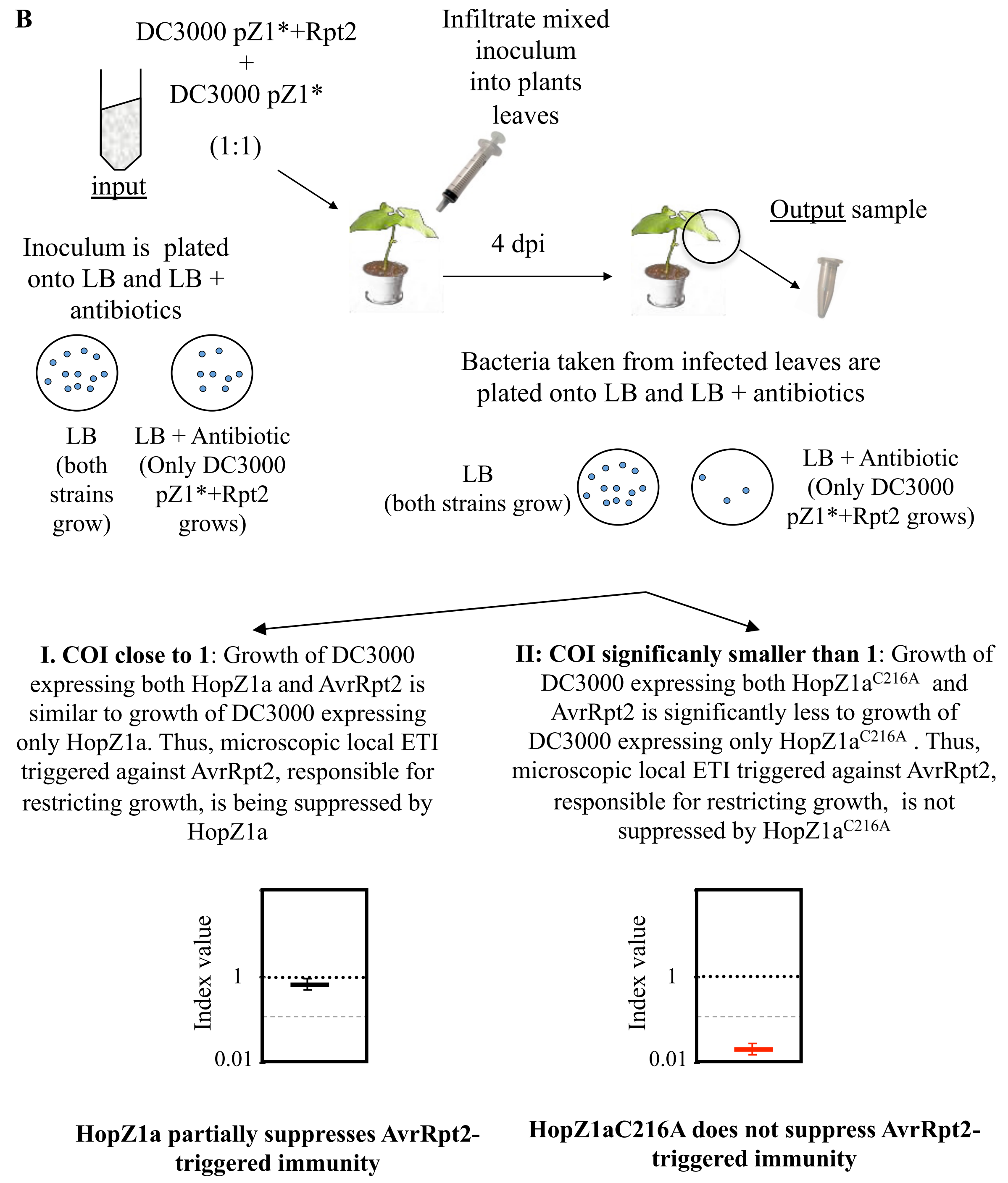

Figure S1. Diagram depicting the cancelled-out analysis carried out in this study. A. COI is defined as ratio between cfu of the the strain expressing both AvrRpt2 and any version of HopZ1a, and the cfu of the strain expressing only the corresponding HopZ1a version in the output sample, divided by their ratio within the input inoculum. **B**. Determination and analysis of COI. A mix inoculum containing an equal bacterial number of both strains is infiltrated into plant leaves. The inoculum is plated onto LB and LB supplemented with antibiotics, to differentiate between the co-inoculated strains, and to establish the input ratio which should be close to 1. Bacteria are recovered from plant leaves at 4 days post inoculation (dpi), and plated into LB and LB supplemented with antibiotics, to differentiate the strains ant to determine their output ration. I and II represent the two control outcomes for the analysis.
