# Supplementary material for: Auto-acetylation on K289 is not essential for HopZ1a-mediated plant defense suppression
Source: Front Microbiol. 2015 Jul 8;6:684. doi: 10.3389/fmicb.2015.00684 (PMC4495678; doi:10.3389/fmicb.2015.00684)
Supplement: Supplementary file 2 [file Image_2.PDF]

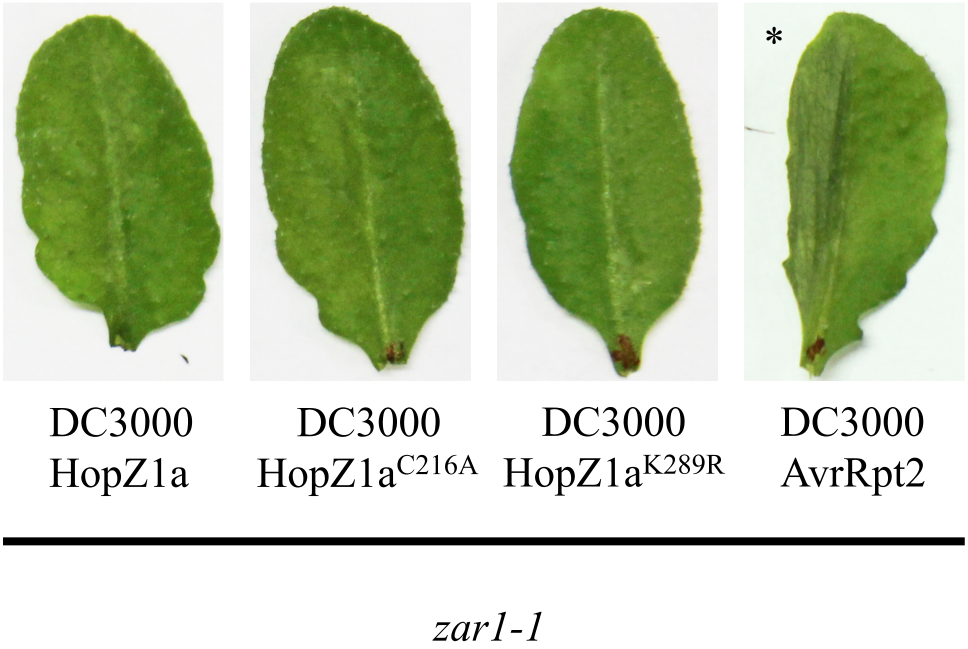

Supplementary Figure 2

Half-leaves of *Arabidopsis zar1-1* inoculated with DC3000 expressing HopZ1a, HopZ1a^{K289R}, or HopZ1a^{C216A} do not display any visible cell death symptoms. Cell death symptoms in response to hand-inoculation of *zar1-1* leaves with bacterial suspensions containing 5 x 10^7 cfu/ml of DC3000, and DC3000 expressing HopZ1a (pAME30), HopZ1a^{C216A} (pAME27), or HopZ1a^{K289R} (pMAM1). DC3000 expressing the ETI-triggering effector AvrRpt2 (pAME8) was included as a control. Photographs were taken 20 hours post-inoculation. Half-leaves displaying cell death symptoms are marked with an asterisk. Images are representative of at least 20 inoculated leaves per strain and experiment.
